# Supplementary material for: Development and ex-vivo assessment of a novel patient specific guide and instrumentation system for minimally invasive total shoulder arthroplasty
Source: PLoS One. 2021 May 21;16(5):e0251880. doi: 10.1371/journal.pone.0251880 (PMC8139503; doi:10.1371/journal.pone.0251880)
Supplement: S1 Appendix — This appendix provides detailed descriptions of the various instruments used in the minimally invasive shoulder replacement procedure and in the case of the patient specific guides, how they are created. (DOCX) [file pone.0251880.s002.docx]

**S1 Appendix.** **Detailed Descriptions of Novel Instruments**

# NOVEL PATIENT SPECIFIC GUIDE (PSG)

In this work, we developed a PSG (Figure S-1) to serve two purposes. First, the PSG guides the drilling of a trans-humeral bone tunnel for use as a guide for humeral bone preparation and as a working channel. Second, the PSG guides drilling of a glenoid guide hole created simultaneously by drilling from the lateral humerus and into the scapula (Figure S-1C). These objectives were achieved by designing a PSG that coaxially aligns the central guide axis of both bones (i.e. the central axis that controls each implant’s position and orientation). The PSG has two opposing contoured surfaces that incorporate unique anatomical features from both the humerus and scapula (Figure S-1). By incorporating features of both bones into one PSG, the guide can lock the bones together in a pre-operatively defined pose (i.e. position and orientation). Additionally, by using this design with a muscle sparing surgical approach, the PSG’s physical registration (i.e. locking) to the bones is assisted by joint compression caused by passive tension in the intact rotator cuff.


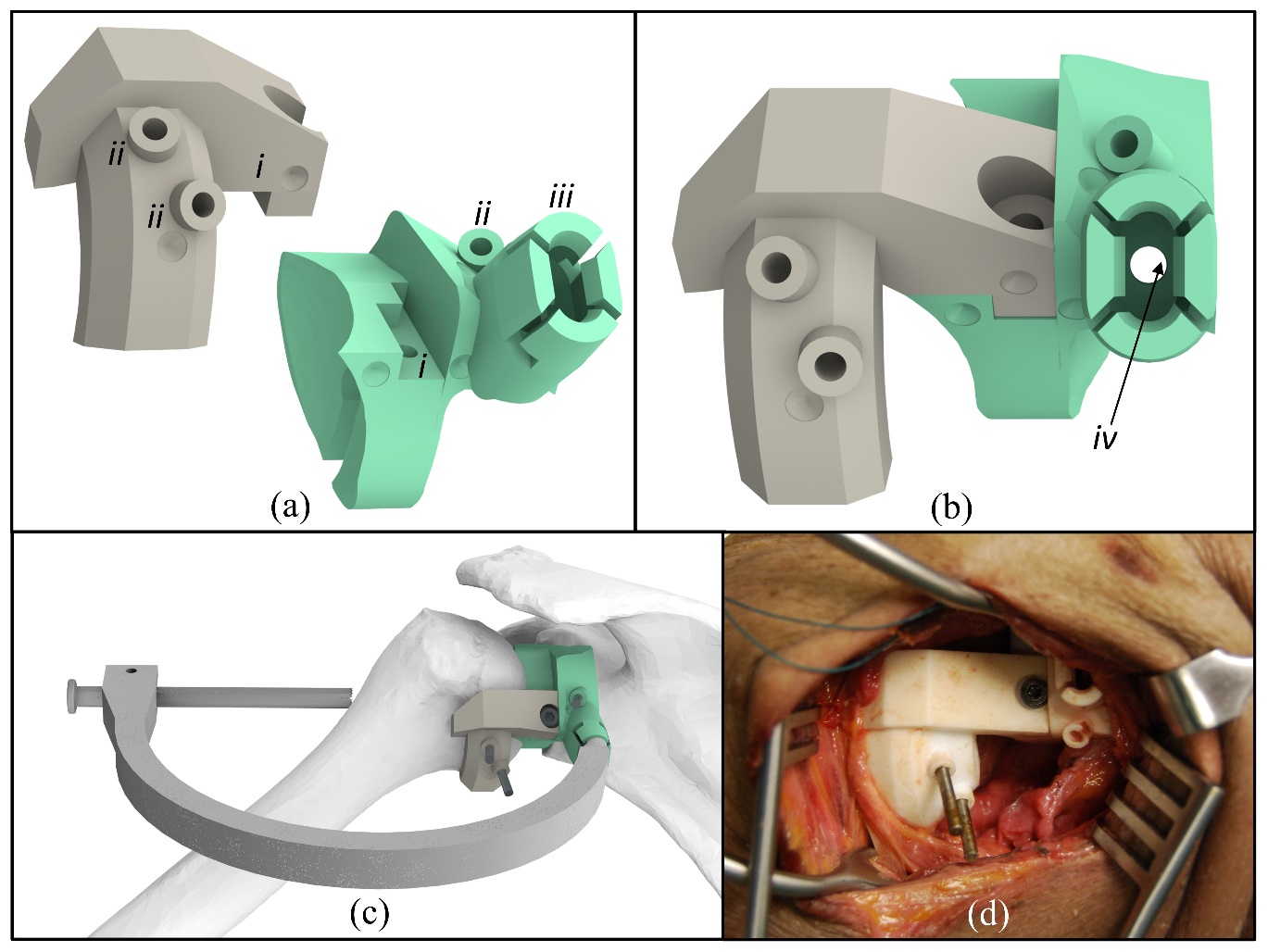


*Figure S-1: Computer renderings and photo of two-sided minimally invasive PSG. (A) Two PSG components from posterior-inferior view before being connected together with critical features including inter-lock mechanism (i), fixation pin holes (ii), and a standardized snap-fit feature for drill guide attachment (iii). (B) Posterior view with components connected showing hole used to guide creation of reference mark on glenoid rim (iv). (C) PSG registered to the humerus and scapula, such that they are placed in a pre-operatively planned pose with attached c-shaped drill guide that enables simultaneous trans-humeral and glenoid guide hole creation, and with fixation pins inserted guides into bone and bolt rigidly fixing guide together, (D) photo showing PSGs registered to bones within posterior muscle splitting surgical approach.*

The PSG is created by first importing CT-derived 3D humerus and scapula models into a computer aided design package (SolidWorks, Dassault Systèmes, Paris, France). The desired central guide axis orientation and articular insertion point are then identified for each bone, and the bones are positioned such that the axes are coaxial. This allows a drill to pass through the humeral head and into the glenoid, creating both guide holes in one step without *en face* intra-articular access. This fully defines the bones’ pose except for the medial-lateral gap between them and their relative flexion-extension rotation, both of which can be adjusted to optimize joint laxity and PSG registration. Specifically, the medial-lateral gap (i.e. PSG thickness) can be adjusted to ensure sufficient passive joint compression is produced when the guide is inserted between the articular surfaces. Additionally, the relative flexion-extension can be adjusted to ensure the PSG overlays the most unique geometry of the humeral neck (e.g. the calcar); this typically corresponds to 0-10° of humeroscapular extension.

The PSG is then designed around the unique geometry of the patient’s posterior glenoid rim and posterior humeral articular margin to achieve robust physical registration (Figure S-1C). The design incorporates a modular humeral component – which eases PSG introduction to the joint and decreases invasiveness – that is registered and pinned to the humerus before being rigidly connected to the PSG’s main body **(**Figure S-1A&B). Furthermore, two versions of the guide’s main body are designed to accommodate the pre-operatively unknown laxity of the patient’s glenohumeral joint. The first is ~3 mm thick between the humeral and glenoid surfaces at the guide axis location and the second is 5 mm thicker. This ensures that a guide is available intra-operatively that produces sufficient passive joint compression to achieve good registration. Finally, a pre-designed snap fit feature is positioned and merged with the posterior surface of the PSG design such that, when a c-shaped drill guide is attached, it enables the guiding holes in each bone to be accurately drilled (Figure S-1). A patent application (GB1504122.1, Priority Date: 11/03/15) has been filed for this novel PSG design. For the purposes of this study, the PSG was fabricated using an industrial quality 3D printer (Formiga P110, EOS Systems, Warwick, UK).

# NOVEL INSTRUMENTATION

*C-Shaped Drill Guide*

To accurately achieve simultaneous trans-humeral drilling, a c-shaped drill guide (Figure S-1C) was designed to rigidly attach to the PSG’s snap fit feature. The offset between the guide’s drilling axis and the intra-articular snap fit feature (23 mm) was chosen to enable its use with a full range of glenoid widths. Finally, to reduce the risk of axillary nerve injury, the guide incorporates a cannula that can be slid through the lateral incision until it contacts the lateral humerus.

*Humeral Cutting Guide*

The Affinis short-stemmed humeral implant used in this study requires humeral head resection; however, the standard technique – estimating retroversion by visualizing the anterior and posterior articular margins – cannot be readily achieved within an MI environment. Therefore, we designed a novel, adjustable cutting guide (Figure S-2) that references the humeral guide hole that is pre-operatively defined to be perpendicular to the humeral resection plane. The cutting guide has three main components: a slotted, saw blade guide block, and two perpendicular shafts that allow the guide to be adjusted to the humeral head size. The shaft attached to the cutting block is parallel to the humeral guide hole and allows the cutting slot to be aligned with the intended cutting plane (e.g. at the articular margin). The second shaft connects to the first by a sliding connector to allow the cutting block to be moved radially until it contacts the humeral head (i.e. adjusting for head diameter). This second shaft has a short 4 mm diameter pin that is inserted into the humeral guide hole thus controlling the cutting block’s orientation.


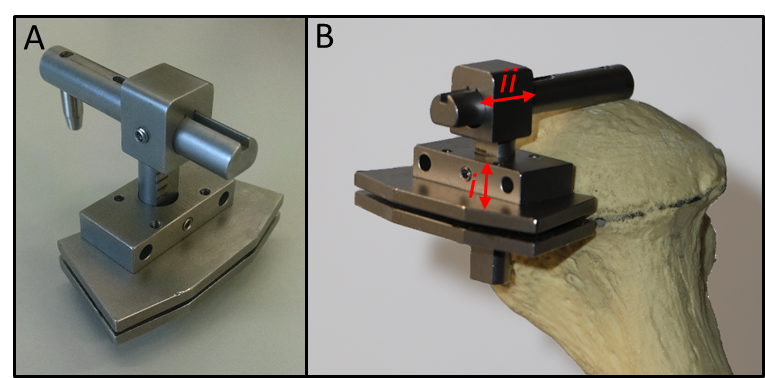


Figure S-2 – (A) Photo of humeral head resection guide with head height adjustability indicated by vertical arrow & width by horizontal arrow, (B) photo of resection guide mounted to foam humerus by guide pin placed in humeral tunnel with adjustment for (i) humeral head cut thickness and (ii) humeral head diameter.

*Glenoid Reamer*

To ream the glenoid within the MI environment, the standard instrumentation and technique were modified in two ways. First, reamers matching the standard Affinis instrumentation were designed but with an added central guide pin to be inserted into the glenoid guide hole (Figure S-3). This modification was used because without dislocating the joint, there is insufficient joint space to use a standard glenoid pin and cannulated reamer. Second, the reamer is powered by a 4 mm diameter driver passed through the trans-humeral bone tunnel and connected to the reamer intra-articularly. Once the reamer and driver are connected, the reamer guide pin can be inserted into the glenoid guide hole and reaming can be conducted while the humerus is lightly retracted to prevent it from contacting the back of the reamer.


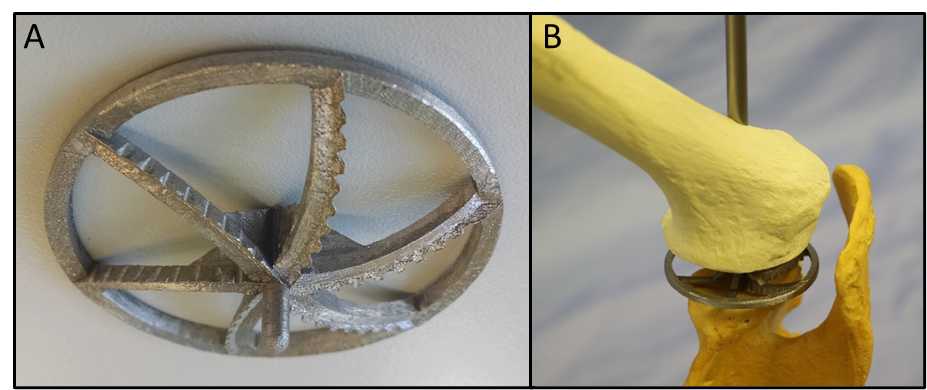


Figure S-3 – (A) Photo of glenoid reamer with integral guide pin, (B) foam bone demonstration of glenoid reamer within the joint and connected to trans-humerally inserted driver.

*Minimally Invasive Off-Axis Drilling Mechanism*

As with most commercial glenoid implants, the Affinis glenoid uses multiple fixation pegs. Drilling holes for these pegs in an MI environment is not possible using traditional methods that require unobstructed perpendicular access to the glenoid surface. Furthermore, right angle drilling tools – similar to a dental drill – are unable to produce the required 8.5 mm diameter peg holes. To overcome this, a novel surgical drilling tool was designed that is powered by a centrally located driver passed through the humerus but which can drill holes that are radially displaced from the driver’s axis. This tool is composed of an epicyclic (i.e. planetary) gear drilling mechanism (Figure S-4) that is additively manufactured from stainless steel in its assembled configuration. It is composed of a six-part assembly (printed in its assembled configuration) and a housing (printed separately) that the mechanism is placed within during surgery. The epicyclic mechanism has a central sun gear (with a mating feature to attach a surgical driver), four symmetrically positioned planet gears, and an outer ring gear. Two of the diametrically opposed planet gears have custom 8.5mm diameter drill bits extending from them that are sufficiently long to drill the implant peg holes. Rotation applied to the sun gear by a surgical driver rotates the planet gears (and attached drill bits), which enables radially displaced drilling. The gears use a double helix (i.e. herringbone) tooth geometry that resists transverse forces and thus the system cannot be disassembled from its additively manufactured configuration; thus, it is an inexpensive, easily manufactured disposable tool. The housing fits around the gear system with the drill bits protruding through its base and constrains the planet gears such that they rotate about their axis without precessing.

The system is powered by a driver that is inserted through the humerus, the sun gear mating feature, the housing, and extends past the drill bit tips such that it can be inserted into the glenoid guide hole. This guide pin controls the drill’s position, version, and inclination, but rotation around the guide axis is controlled by the surgeon using a handle attached to the drill housing (Figure S-4). The handle orientation, and thus rotation around the driver axis of the holes drilled by the mechanism, is guided by aligning it to a reference mark on the posterior glenoid vault. The reference mark is created while the PSG is fixed to the scapula using a drill bit and guide hole on the PSG itself (Figure S-1.iv). The guide hole is positioned on the PSG such that the glenoid reference mark it creates will yield peg holes matching the pre-operative plan. The mechanism is operated by powering the driver, using the handle to control rotation, and advancing the mechanism until the base of the housing contacts the reamed glenoid which will produce the correct peg hole depth.


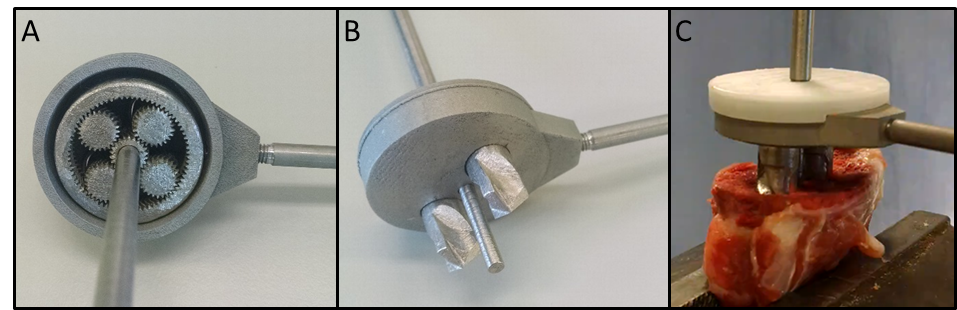


Figure 4 – (A) Photo of epicyclic gear system with driver inserted through sun gear, and rotation control handle affixed to housing, (B) photo of drill bits connected to two opposing planet gears, and extension of driver through housing to be inserted into glenoid guide hole, (C) photo of drilling mechanism in use during an ex-vivo trial.

*Glenoid Implant Impaction*

To enable the glenoid implant to be safely impacted without dislocating the joint, a c-shaped impactor was designed that avoids the humeral head while allowing a contoured impaction head to be inserted through the posterior incision. This design ensures that when the impactor is struck by a surgical mallet only forces parallel to the pegs are transmitted to the implant.

*Humeral Impaction*

Two options were developed to impact the humeral stem and ceramic head: 1) a traditional impactor for cases where direct impaction is possible, and 2) a slap hammer impactor for cases where there was insufficient internal rotation to allow direct impaction (Figure S-5). The slap hammer was modularly designed with a c-shaped component (similar to the glenoid impactor) that allowed standard Mathys cutters and compactors to be attached to one end and inserted into the joint. At the other end of the c-shaped component a guide pin is inserted into the lateral entrance of the humeral bone tunnel and attached to the impactor to ensure forces are applied parallel to the humeral guide axis. Once the pin is attached, a slap hammer shaft and mass are attached and the cutters, compactors, final stem, and humeral head trial and final component can be sequentially impacted.


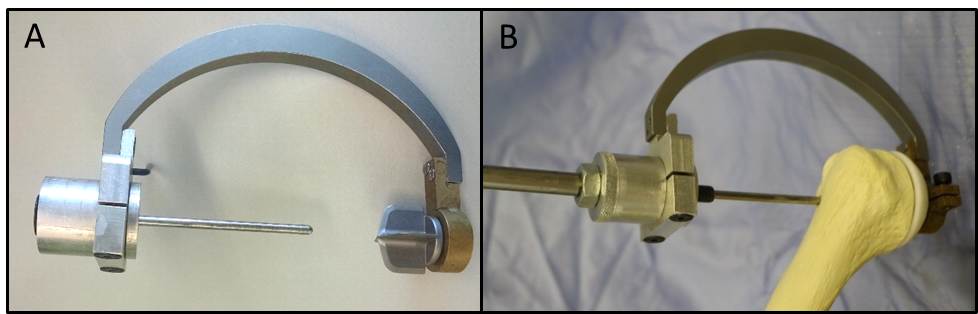
Figure S-5 – (A) Photo of the humeral slap hammer impactor with guide pin and cruciform cutter in place, (B) photo of slap hammer assembled onto humerus and configured to impact final humeral head implant.
