# Supplementary material for: Development and ex-vivo assessment of a novel patient specific guide and instrumentation system for minimally invasive total shoulder arthroplasty
Source: PLoS One. 2021 May 21;16(5):e0251880. doi: 10.1371/journal.pone.0251880 (PMC8139503; doi:10.1371/journal.pone.0251880)
Supplement: S2 Appendix — This appendix provides details on how the accuracy of each surgical step in the procedure was measured and assessed. (DOCX) [file pone.0251880.s003.docx]

**S2 Appendix: Processes for Assessing Accuracy of Intermediate Steps**

*Accuracy of Intermediate Steps*

The accuracy of all intermediate surgical steps was assessed by transforming digitized experimental points and comparing them to the pre-operative plan in terms of translation and/or rotation. To assess the positional accuracy of the drilled guide holes, the point at which the guide holes penetrated the articular surface of each bone was digitized, transformed, and compared to the pre-operative plan. Additionally, the point where the humeral guide hole exited the lateral cortex as well as the deepest point of the guide hole in the glenoid vault were also digitized. These additional points were combined with the articular points for each guide hole to create drill orientation lines, and these were used to calculate the 3D rotational error compared to the pre-operative plan. To assess humeral head resection accuracy, a continuous trace of the resection plane was digitized, all of the points were transformed, the normal vector to the least squares best fit plane was computed, and the 3D angular error compared to the planned humeral resection normal vector was determined.
